# The Randomized Controlled STRAWINSKI Trial: Procalcitonin-Guided Antibiotic Therapy after Stroke

**DOI:** 10.3389/fneur.2017.00153

**Published:** 2017-04-24

**Authors:** Lena Ulm, Sarah Hoffmann, Darius Nabavi, Marcella Hermans, Bruno-Marcel Mackert, Frank Hamilton, Ingo Schmehl, Gerhard-Jan Jungehuelsing, Joan Montaner, Alejandro Bustamante, Mira Katan, Andreas Hartmann, Stefan Ebmeyer, Christiane Dinter, Jan C. Wiemer, Sabine Hertel, Christian Meisel, Stefan D. Anker, Andreas Meisel

**Affiliations:** ^1^NeuroCure Clinical Research Center, Charité – Universitaetsmedizin Berlin, Berlin, Germany; ^2^Centre for Clinical Research, The University of Queensland, Brisbane, QLD, Australia; ^3^Department of Neurology and Center for Stroke Research Berlin, Charité – Universitaetsmedizin Berlin, Berlin, Germany; ^4^Department of Neurology, Vivantes Klinikum Neukoelln, Berlin, Germany; ^5^Department of Neurology, Vivantes Auguste-Viktoria-Klinikum, Berlin, Germany; ^6^Department of Neurology, Unfallkrankenhaus Berlin, Berlin, Germany; ^7^Department of Neurology, Juedisches Krankenhaus Berlin, Berlin, Germany; ^8^Neurovascular Research Laboratory, Institut de Recerca, Hospital Universitari Vall d’Hebrón, Universitat Autònoma de Barcelona, Barcelona, Spain; ^9^Department of Neurology, Universitaetsspital Zuerich, Zurich, Switzerland; ^10^Department of Neurology, Klinikum Frankfurt Oder, Frankfurt Oder, Germany; ^11^Thermo Fisher Scientific BRAHMS GmbH, Hennigsdorf, Germany; ^12^Department of Immunology, Charité – Universitaetsmedizin Berlin, Berlin, Germany; ^13^Division of Innovative Clinical Trials, Department of Cardiology and Pneumology, University Medical Centre Goettingen, Goettingen, Germany; ^14^Centre for Clinical and Basic Research, IRCCS, Rome, Italy

**Keywords:** stroke, pneumonia, antibiotic prophylaxis, procalcitonin, outcome, infections, biomarker-guided treatment

## Abstract

**Background:**

Pneumonia is among the most common acute complications after stroke and is associated with poor long-term outcome. Biomarkers may help identifying stroke patients at high risk for developing stroke-associated pneumonia (SAP) and to guide early treatment.

**Aims:**

This trial investigated whether procalcitonin (PCT) ultrasensitive (PCTus)-guided antibiotic treatment of SAP can improve functional outcome after stroke.

**Methods:**

In this international, multicenter, randomized, controlled clinical trial with blinded assessment of outcomes, patients with severe ischemic stroke in the middle cerebral artery territory were randomly assigned within 40 h after symptom onset to PCTus-based antibiotic therapy guidance in addition to stroke unit care or standard stroke unit care alone. The primary endpoint was functional outcome at 3 months, defined according to the modified Rankin Scale (mRS) and dichotomized as acceptable (≤4) or unacceptable (≥5). Secondary endpoints included usage of antibiotics, infection rates, days of fever, and mortality. The trial was registered with http://ClinicalTrials.gov (Identifier NCT01264549).

**Results:**

In the intention-to-treat-analysis based on 227 patients (112 in PCT and 115 in control group), 197 patients completed the 3-month follow-up. Adherence to PCT guidance was 65%. PCT-guided therapy did not improve functional outcome as measured by mRS (odds ratio 0.79; 95% confidence interval 0.45–1.35, *p* = 0.47). Pneumonia rate and mortality were similar in both groups. Days with fever tended to be lower (*p* = 0.055), whereas total number of days treated with antibiotics were higher (*p* = 0.004) in PCT compared to control group. A *post hoc* analysis including all PCT values in the intention-to-treat population demonstrated a significant increase on the first day of infection in patients with pneumonia and sepsis compared to patients with urinary tract infections or without infections (*p* < 0.0001).

**Conclusion:**

PCTus-guided antibiotic therapy did not improve functional outcome at 3 months after severe ischemic stroke. PCT is a promising biomarker for early detection of pneumonia and sepsis in acute stroke patients.

## Introduction

Infections are among the most common acute complications after stroke and associated with poor outcome (1, 2). Prophylactic antimicrobial treatment effectively reduced infection rates in experimental models of stroke (3) and clinical proof-of-concept studies [for a meta-analysis, see the study by Westendorp et al. (4)]. However, the impact of preventive antibiotic treatment on long-term functional outcome remained unclear, as previous clinical trials were not sufficiently powered to address this issue. Recently, two large randomized controlled phase III clinical trials demonstrated that antibiotics commonly used to treat stroke-associated pneumonia (SAP) neither reduce the frequency of pneumonia nor improve the outcome after stroke when administered in a prophylactic manner (5–7).

Current European and US stroke guidelines strictly recommend early antibiotic treatment of poststroke infections but advise against their prophylactic use (8). Biomarkers might help to identify patients in the early subclinical course of SAP or even at high risk for developing SAP, thereby tailoring antibiotic treatment to patients with the highest probability to benefit while reducing the risk of antibiotic resistances (9). Procalcitonin (PCT), an early marker of severe bacterial infections (10), has been useful in diagnosing SAP in previous observational clinical studies (11, 12). In addition, PCT guidance has been shown to reduce the duration of antibiotic treatment in critically ill patients, without compromising patients’ safety. In this study, we investigated whether PCT ultrasensitive (PCTus)-guided antibiotic treatment improves functional outcome after severe stroke by early identification and treatment of pneumonia.

## Methods

### Study Design and Participants

Patients from 10 study centers were randomly assigned in a 1:1 ratio to standard stroke unit care plus PCTus-guided antibiotic treatment or to standard stroke unit care alone. Study procedures have been described in detail elsewhere (13). Inclusion criteria were age ≥18 years, severe ischemic stroke (score of >9 on the National Institute of Health Stroke Scale), and clinical diagnosis of a stroke in the middle cerebral artery (MCA) territory. In the initial study protocol, enrollment was planned within 36 h after symptom onset. As this time window turned out to be logistically difficult for the PCT measurements, we amended the study protocol and changed it to <40 h after symptom onset. Exclusion criteria included CT/MRI evidence of intracerebral hemorrhage or lacunar infarction, use of antibiotics within the last 10 days, suspected life expectancy of <3 months (irrespective of the underlying cause), modified Rankin Scale (mRS) before stroke onset ≥4, participation in other interventional trials (pharmaceuticals or medical devices), and pregnancy/lactation.

### Standard Protocol Approvals, Registrations, and Patient Consents

The trial was registered with http://ClinicalTrials.gov (Identifier NCT01264549). Patients’ safety was ensured by daily clinical monitoring throughout the intervention period, as well as severe adverse event (SAE) documentation, and supervision by a Data Safety Monitoring Board (13).

### Randomization and Masking

Creation of randomization lists and treatment allocations were described previously (13). Local investigators were not masked, but patients and assessors of outcome were masked to which group the patients were assigned to. Outcome after 3 months was centrally assessed by structured telephone interviews conducted by trained staff members in the study center. Outcomes after 6 months were assessed by personal anamnesis and medical records. If these were not available, telephone interviews were conducted as for the 3-month outcome assessment. Where recontacting was not successful, an enquiry was sent to the registration office where possible to establish if the patients were still alive.

### Intervention

#### Control Group

Patients were treated according to the current standards of therapy. With respect to antibiotics, they were fully and effectively treated as soon as an antibiotic medication was indicated, i.e., as soon as an infection was diagnosed by the treating physician based on current guidelines (8).

#### PCT Group

PCT ultrasensitive was assessed at study inclusion and every morning during routine clinical evaluation for 7 days. In patients with PCTus concentration ≤0.05 ng/ml, a bacterial infection was considered unlikely, and the use of antibiotics was discouraged. In patients with a PCTus concentration >0.05 ng/ml, a bacterial infection was considered likely, and the use of antibiotics was recommended. As in the control group, type and duration of antibiotic treatment were left to the discretion of the treating physician. The rationale for choosing a cutoff value of 0.05 ng/ml has been described previously (13).

### Outcomes

The primary endpoint was functional outcome 3 months after stroke onset measured by mRS, dichotomized as acceptable outcome (≤4) or unacceptable outcome (≥ 5) (13). In line with previous trials that included patients with severe MCA strokes (14), this dichotomization was chosen because survival without or with only mild disability following severe MCA infarction is rare. The primary aim of this study was to investigate whether PCTus-guided antibiotic treatment reduces mortality without increasing the number of severely disabled survivors (mRS score of 5). Secondary endpoints included time to first event of death or rehospitalization; mortality, infection rate, antibiotic use, and days with fever up to day 7; shift analysis of the mRS; Barthel Index (BI) at day 90; and mRS and BI at day 180.

### Statistical Analyses and Sample Size Calculation

The aim of this study was to estimate the probability of an acceptable outcome (mRS 0–4) at day 90, as well as the proportion of patients receiving antibiotic treatment for both the new regime (PCT group) and the standard treatment (control group) based on the intention-to-treat (ITT) population. Two scenarios were considered a positive result: (a) improved outcome in the PCT arm, while the usage of antibiotics is equivalent, or (b) reduced usage of antibiotics in the PCT arm, while the outcome remains equivalent. A sample size of 70–90 per arm was considered to be sufficient to estimate each individual group proportion for both endpoints with a precision of <0.1 [95% confidence interval (CI)] and a power of 75–80% (13). To account for possible dropouts, a sample size of 100 per arm was targeted. In October 2013, we amended the protocol to increase the sample size to 230 due to a 20% rate of missing outcomes at the 3-month assessment and without knowledge of any of the outcomes at this time.

Patients were included in the per-protocol (PP) analysis, if they were treated according to the study protocol (which allowed overruling of the PCT guidance) (13), whereas the PP^adherence^ analysis only included patients who had been treated strictly according to PCT guidance. Statistical associations were analyzed using chi-square or Fisher’s exact tests (for less than 5 in one category) for categorical variables. Group comparisons of continuous variables were analyzed by Wilcoxon rank-sum test (skewed distribution) or *t*-test (symmetrical distribution). Statistical significance was set at *p* < 0.05. The mRS was additionally analyzed as a continuous instead of a categorical variable to include the full range of this variable and to avoid loss of information by dichotomization. The shift analysis was performed by a randomization approach according to Howard et al. (15), to achieve a more detailed comparison of the frequency distributions of mRS 3-month outcomes between PCT and standard groups. This analysis was performed for the entire ITT patient population, as well as for patient strata with either initial mRS 4 or mRS 5 to account for imbalance in the initial stroke severity between the groups.

## Results

**Figure 1:**
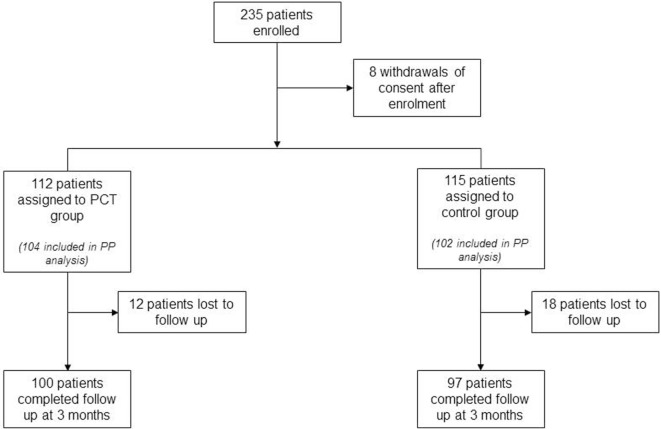
**Trial profile**.

Between February 2011 and April 2014, 235 patients were enrolled and randomly assigned to the 2 study groups. Eight patients withdrew consent after study enrollment but before the first study-related assessment was performed, resulting in a total of 227 patients for the ITT analysis (112 in the PCT group and 115 in the control group). Two hundred six patients (104 in the PCT group and 102 in the control group) were included in the PP analysis. Adherence to PCT guidance was 65%, leaving 170 patients for the PP^adherence^ analysis (68 in the PCT group and 102 in the control group). Physicians overruled PCT guidance either by treating with antibiotics despite PCT values below the cutoff (28% of patients in the PCT group) or by not treating patients with antibiotics although PCT values were above the cutoff value indicative of an underlying bacterial infection (37% of patients in the PCT group). Eight centers included more than 10 patients into this study. Among these, the adherence to PCT guidance ranged between 33 and 100% (mean 60%). The 3-month follow-up was completed by 197 (87%) patients from ITT group. The rate of 3-month follow-ups was 84% (*n* = 97) in the control group and 89% (*n* = 100) in the PCT group (Figure 1; *p* = 0.28).

The study groups did not differ regarding baseline demographic and clinical characteristics, except for a higher rate of chronic obstructive pulmonary disease (COPD) in the PCT group and a higher rate of diabetes mellitus in the control group (Table 1). There was a trend toward higher baseline mRS scores at the time of the initial study visit in the control group (*p* = 0.067 in ITT; *p* = 0.070 in PP).

**Table 1:** **Baseline characteristics by analysis group (ITT and PP)**.

Variable	Level	ITT			PP		
		PCT (*N* = 112)	STD (*N* = 115)	*p* Value	PCT (*N* = 104)	STD (*N* = 102)	*p* Value
Gender, *N* (%)	Male	52 (46)	49 (43)	0.66	50 (48)	44 (43)	0.57
	Female	60 (54)	66 (57)		54 (52)	58 (57)	
Age (years), mean (SD)		76.2 (11.5)	76.1 (11.3)	0.91	76.1 (11.8)	75.3 (11.6)	0.63
Total NIHSS initial visit, median (IQR)		14 (12–18)	15 (12–19)	0.49	14 (11.75–17)	14 (12–18)	0.53
Barthel Index at day 7, median (IQR)		15 (0–40)	10 (0–33.75)	0.21	15 (0–40)	10 (0–35)	0.24
mRS before study, *N* (%)	0	61 (55)	72 (64)	0.46	59 (57)	66 (66)	0.42
	1	17 (15)	18 (16)		15 (14)	15 (15)	
	2	11 (10)	8 (7)		10 (10)	8 (8)	
	3	21 (19)	13 (12)		18 (17)	11 (11)	
	4	2 (2)	1 (1)		2 (2)	0 (0)	
mRS at initial visit, *N* (%)	≤4	44 (39)	31 (27)	0.067	42 (40)	28 (28)	0.07
	5	68 (61)	84 (73)		62 (60)	74 (73)	
mRS at day 7, *N* (%)	1	3 (3)	0 (0)	0.68	3 (3)	0 (0)	0.60
	2	5 (5)	4 (4)		5 (5)	4 (4)	
	3	9 (8)	8 (7)		8 (8)	8 (8)	
	4	33 (30)	38 (34)		33 (32)	36 (35)	
	5	54 (50)	57 (50)		52 (51)	54 (53)	
	6	5 (5)	6 (5)		1 (1)	0 (0)	
Diabetes mellitus, *N* (%)	Yes	24 (22)	38 (34)	0.08	22 (22)	36 (36)	0.048
	No	84 (78)	75 (66)		78 (78)	65 (64)	
Atrial fibrillation, *N* (%)	Yes	55 (51)	59 (53)	0.96	52 (53)	51 (51)	0.94
	No	52 (49)	53 (47)		47 (48)	49 (49)	
History of stroke, *N* (%)	Yes	31 (29)	23 (21)	0.26	27 (27)	21 (21)	0.45
	No	77 (71)	86 (79)		73 (73)	77 (79)	
Hypertension, *N* (%)	Yes	94 (85)	99 (87)	0.79	87 (85)	89 (87)	0.71
	No	17 (15)	15 (13)		16 (16)	13 (13)	
Hypercholesterolemia, *N* (%)	Yes	54 (51)	61 (55)	0.70	51 (52)	54 (54)	0.95
	No	52 (49)	51 (46)		47 (48)	47 (47)	
Coronary heart disease, *N* (%)	Yes	22 (22)	30 (28)	0.40	22 (24)	27 (28)	0.62
	No	78 (78)	77 (72)		70 (76)	69 (72)	
COPD, *N* (%)	Yes	19 (19)	6 (6)	0.007	19 (20)	5 (5)	0.005
	No	83 (81)	100 (94)		75 (80)	89 (95)	
Smoker, *N* (%)	Yes	18 (19)	13 (13)	0.30	18 (21)	13 (14)	0.33
	No	76 (81)	89 (87)		68 (79)	78 (86)	
Thrombolysis, *N* (%)	Yes	61 (54)	54 (47)	0.26	58 (56)	48 (47)	0.21
	No	51 (46)	61 (53)		46 (44)	54 (53)	

*COPD, chronic obstructive pulmonary disease; IQR, interquartile range; mRS, modified Rankin Scale; NIHSS, National Institute of Health Stroke Scale; ITT, intention to treat; PP, per protocol; PCT, procalcitonin group; STD, control group*.

*p Values are calculated by Fisher’s test, chi-square test, Wilcoxon test, or t-test as appropriate*.

Apart from SAP, the frequency of SAEs was low. There was no significant difference in the frequency of SAEs between the study groups (Table 2).

**Table 2:** **Serious adverse event reports per study group for intention-to-treat analysis (*N* = 227)**.

	PCT (*N* = 112), *N* (%)	STD (*N* = 115), *N* (%)
Pneumonia	33 (29)	31 (27)
Death	4 (4)	6 (5)
Pulmonary embolism	1 (1)	2 (2)
Sepsis	2 (2)	1 (1)
Intracerebral hemorrhage	2 (2)	0 (0)
Heart failure	1 (1)	2 (2)
Acute myocardial infarction	2 (2)	1 (1)
Reinfarction	0 (0)	3 (3)

*PCT, procalcitonin group; STD, control group*.

*Data are based on day 1–7 of the study*.

**Figure 2:**
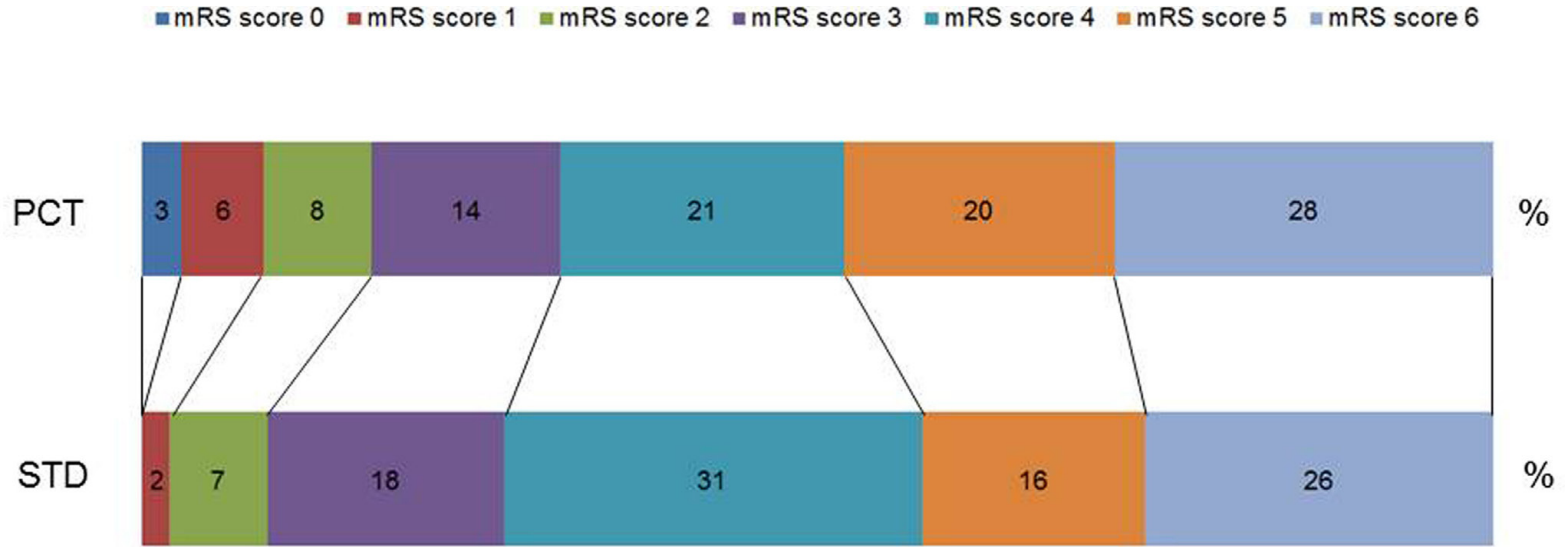
**Distribution of modified Rankin Scale (mRS) scores 3 months after stroke onset**. Percentage of respective scores in the guidance [procalcitonin (PCT)] group and control group (STD) in the intention-to-treat analysis. Scores on the scale range from 0 to 6, with 0 indicating no symptoms and 6 indicating death.

**Figure 3:**
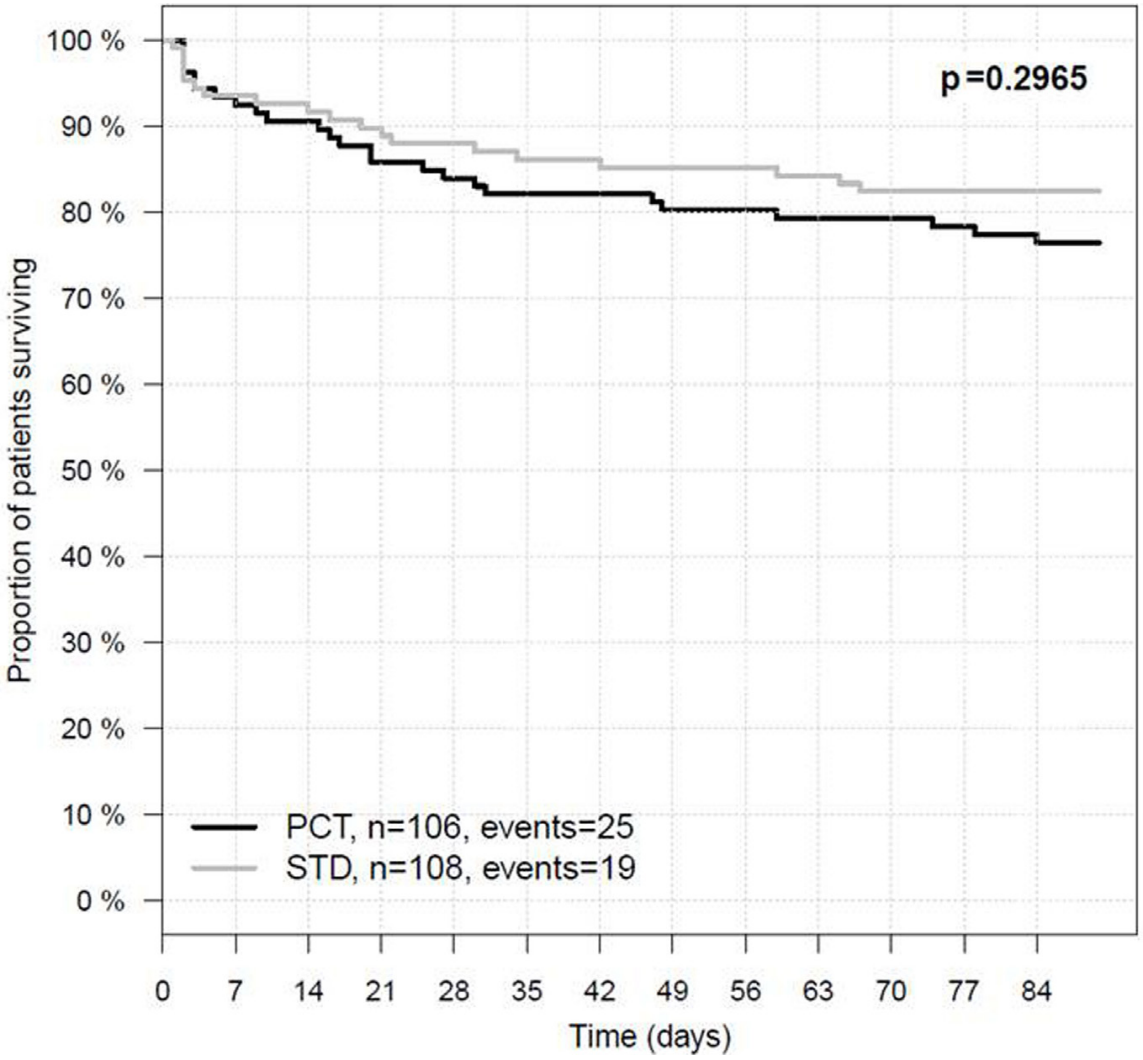
**Three-month survival in the intention-to-treat population**. PCT, PCT group; STD, control group. In the PCT group, 81 of 106 patients survived 90 days after stroke onset, whereas in the STD group, 89 of 108 patients survived.

Procalcitonin-based antibiotic therapy guidance was not associated with an increased proportion of acceptable outcome (mRS ≤ 4) at 3 months, neither in the ITT (OR 0.79, 95% CI 0.45–1.39, *p* = 0.47) nor in the PP (OR 0.71, 95% CI 0.39–1.48, *p* = 0.29) or PP^adherence^ analyses (OR 0.81, 95% CI 0.42–1.57, *p* = 0.61, Table 3). The mRS distribution 3 months after stroke onset in both study groups is shown in Figure 2. In secondary endpoint analyses, outcome at 6 months was similar between both groups (*p* = 0.66 for ITT population). A shift analysis of the mRS (15) also revealed no significant differences between the groups. In all shift analysis scenarios considered, the proportion of better outcome was lower than the proportion of worse outcome for patients of the PCT group when compared with patients of the control group (Table 4). The proportion of a BI of ≥60 in the 3-month follow-up as a measure for favorable outcome was similar in the PCT and control groups (median BI, ITT: 62.5 in PCT group vs. 55 in control group, *p* = 0.415; PP: 62.5 in PCT group vs. 55 in control group, *p* = 0.542). Mortality rates (Figure 3) were not significantly different between groups during 3 months (PCT group vs. control group—ITT: OR 1.20, 95% CI 0.65–2.24; PP: OR 1.28, 95% CI 0.64–2.58) and 6 months of follow-up (PCT vs. control group—ITT: OR 1.34, 95% CI 0.75–2.41; PP: OR 1.42, 95% CI 0.75–2.6).

**Table 3:** **mRS scores at the 3-month follow-up assessment**.

	Group	mRS ≤ 4, *n* (%)	Odds ratio PCT vs. STD	95% CI		*p* Value
				Lower CI	Higher CI	
ITT (*N* = 227)	PCT	52 (52)	0.79	0.45	1.35	0.475
	STD	56 (58)				
PP (*N* = 206)	PCT	50 (54)	0.71	0.39	1.28	0.288
	STD	54 (63)				
PP adh. (*N* = 170)	PCT	37 (58)	0.81	0.42	1.57	0.613
	STD	54 (63)				

*ITT, intention to treat; PP, per protocol; PP.adh., per protocol adherence; PCT, procalcitonin group; STD, control group; CI, confidence interval; mRS, modified Rankin Scale*.

*p Values are calculated by Fisher’s exact test*.

**Table 4:** **Shift analysis of mRS scores at the 3-month follow-up assessment**.

	Shift for mRS V90	Median (IQR)		*p* Value	Randomization approach^a^			
		PCT	STD		Proportion better (%)	Proportion worse (%)	Proportion same (%)	Ratio (better vs. worse)
ITT	All (no adjustment)	4 (3.0–6.0)	4 (3.0–6.0)	0.452	37	42	21	0.88
	mRS V0 = 4	4 (2.5–3.0)	3 (3.0–4.5)	0.477	39	42	19	0.92
	mRS V0 = 5	5 (4.0–6.0)	4 (4.0–6.0)	0.840	30	45	25	0.66
PP	All (no adjustment)	4 (3.0–5.0)	4 (3.0–5.0)	0.563	37	43	20	0.86
	mRS V0 = 4	4 (2.3–4.8)	3 (3.0–4.0)	0.511	38	43	19	0.89
	mRS V0 = 5	5 (4.0–6.0)	4 (4.0–5.0)	0.902	29	48	23	0.62
PP adh.	All (no adjustment)	4 (3.0–5.0)	4 (3.0–5.0)	0.330	40	40	20	0.99
	mRS V0 = 4	3 (2.0–4.0)	3 (3.0–4.0)	0.153	47	33	21	1.45
	mRS V0 = 5	5 (4.0–6.0)	4 (4.0–5.0)	0.873	29	48	23	0.62

*ITT, intention to treat; PP, per protocol; PP.adh., per protocol adherence; mRS, modified Rankin Scale; PCT, procalcitonin group; STD, control group*.

*Results were analyzed stratified by baseline mRS (mRS V0 = 4, mRS V0 = 5) because this variable was distributed differently in both study groups*.

*^a^Based on the study by Howard et al. (15)*.

**Figure 4:**
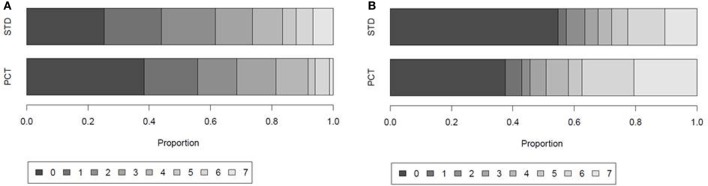
**Days with fever and antibiotic treatment within the first 7 days after stroke onset**. Proportion of patients with 0, 1, 2, 3, 4, 5, 6, or 7 days of **(A)** fever defined by body temperature ≥37.5°C and **(B)** antibiotic treatment in the guidance [procalcitonin (PCT)] group and control group (STD) of the intention-to-treat (ITT) population.

Within the first week after stroke onset, the presence of SAP or sepsis (with or without further infections) was diagnosed in 64 (28%) patients. Urinary tract infection (UTI) without SAP was diagnosed in 19 (8%) patients. Infection rates within the first 7 days after stroke onset were similar in both study groups (SAP 29% in PCT group vs. 27% in STD group; UTI 7% in PCT group vs. 10% in STD group; Table 5).The median number of days with fever tended to be higher in controls (2 days in control group vs. 1 day in PCT group, *p* = 0.055; Figure 4A).

**Table 5:** **Infections and antibiotic treatment per study group for intention-to-treat analysis (*N* = 227)**.

	PCT (*N* = 112), *N* (%)	STD (*N* = 115), *N* (%)	*p* Value
Pneumonia or sepsis	33 (29)	31 (27)	0.79
Urinary tract infection only	8 (7)	11 (10)	0.68
Antibiotic treatment	70 (63)	52 (45)	0.01

*PCT, procalcitonin group; STD, control group. Data are based on day 1–7*.

*p Values are calculated by chi-square test*.

In the ITT analysis, antibiotic treatment was more frequent in the PCT compared to the standard group. Sixty-three percent of patients in the PCT group received antibiotic treatment, compared to 45% of patients in the control group (Table 5). The total number of days treated with (any) antibiotics within the first week after stroke onset was 362 [median 3, interquartile range (IQR) 0–6] in the PCT group (*n* = 112) compared to 250 (median 0, IQR 0–5) in the control group (*n* = 115) (*p* = 0.004; Figure 4B). A *post hoc* analysis of the use of antibiotic substances between day 1 and day 7 after stroke onset showed significant differences between the study groups (Table 6; *p* = 0.0004).

**Table 6:** **Treatment days with different antibiotic classes per study group**.

Group	Antibiotic class						
	Penicillin	Cephalosporin	Quinolone	Macrolide	Lincosamide	Others	Sum
PCT, *N* (%)	174 (35)	148 (30)	17 (3)	34 (7)	16 (3)	109 (22)	498 (100)
STD, *N* (%)	107 (30)	97 (27)	32 (9)	24 (7)	28 (8)	68 (19)	356 (100)

*PCT, procalcitonin group; STD, control group*.

*Days with antibiotic treatment between visit 1 and 7*.

In contrast to patients with UTIs, SAP patients have a mortality rate that was twice as high as in patients without SAP within 3 months of follow-up (Table 7; *p* = 0.0055). Pneumonia rates following the acute course of stroke (3-month follow-up: 6% PCT vs. 7% STD, 6-months follow-up: 8% PCT vs. 9% STD) and rehospitalization rates (3-month follow-up: 10% PCT vs. 7% STD, 6-months follow-up: 14% PCT vs. 10% STD) were comparable in both groups.

**Table 7:** **Three-month mortality stratified by stroke-associated pneumonia and treatment group**.

	Pneumonia or sepsis	No pneumonia and no sepsis
PCT	39% (13 of 33)	20% (15 of 75)
STD	36% (11 of 31)	18% (14 of 80)
Sum	38% (24 of 64)	19% (29 of 155)
*p* Value	0.95	0.69

*PCT, procalcitonin group; STD, control group*.

*% mortality at 3 months (# dead of *N*). *p* Values are calculated by chi-square test*.

**Figure 5:**
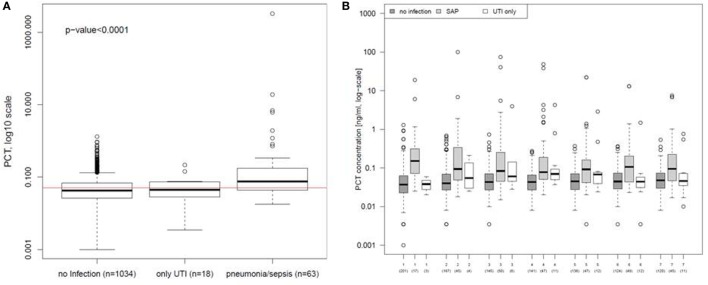
**Procalcitonin (PCT) in different types of poststroke infections**. **(A)** PCT values of the first day of urinary tract infection (UTI) only or stroke-associated pneumonia (SAP) were compared to all PCT values obtained within the first 7 days after stroke onset from patients before infection was diagnosed or without any infection. The red line indicates the cut-off for the PCT concentration of 0.05 ng/ml. **(B)** PCT values of the first 7 days were compared between patients diagnosed for UTI only, SAP, and for patients without any diagnosed infection. Analyses are based on the intention-to-treat group.

A *post hoc* analysis of all PCT values in the ITT population demonstrated a significant increase on the first day of infection in patients with pneumonia and sepsis compared to patients with UTIs or without infections [median (IQR): no infection 0.042 (0.026–0.068), UTI only 0.044 (0.029–0.072), and pneumonia/sepsis 0.076 (0.043–0.175); Figure 5A]. PCT values were significantly higher in patients with SAP or sepsis (±UTI) between day 1 and 7 following stroke onset compared to patients without infections (Figure 5B).

## Discussion

PCT ultrasensitive-guided antibiotic treatment of bacterial pneumonia in acute stroke patients is a safe treatment strategy. However, our study failed to demonstrate an improvement of stroke outcome for this treatment approach. Moreover, SAP and mortality rates were similar in both study groups.

Only recently, two large randomized controlled phase III trials demonstrated neither an improvement of stroke outcome nor a significant reduction of SAP rate by preventive antibiotic therapy (5, 6). Both trials reported a significant reduction in UTIs, which are known to be unrelated to poststroke outcome (7).

In our study, PCTus guidance did not affect the frequency of clinical diagnosis of SAP, since infection rates were similar in both treatment groups. However, the usage of antibiotics (measured in days with antibiotic treatment) was higher in the PCT compared to the control group. Days with fever tended to be reduced by PCTus-guided treatment, which is important to note, since fever is a negative prognostic factor for stroke outcome (16).

Procalcitonin testing has also been shown to improve diagnostic accuracy for SAP in previous observational studies, where it was shown to improve specificity in case of clinical suspicion of SAP (12). Accordingly, a *post hoc* analysis of PCT values in our study demonstrated a significant increase of PCT in patients with SAP and sepsis on the first day of infection, compared to patients with UTIs or without infections. Although there is a frequent request for biomarkers as a criterion for diagnosing SAP (9, 17), the adherence to the PCT guidance was surprisingly low. In the PCT group, overruling of the PCT guidance by the treating physician was allowed due to ethical concerns. Strict guidance to PCT was followed in only 65% of the patients, with considerable differences between the centers. This may be considered a problem of trust by some physicians in this new treatment approach under testing and may have impacted to some degree the trial outcome.

In clinical routine, physicians diagnose SAP based on clinical criteria, mainly fever, and are influenced by risk factors for SAP, e.g., stroke severity, while biomarkers are only used as additional diagnostic information (17). Overall, clinicians tend to overdiagnose SAP (7), reinforcing the need for operational diagnostic SAP criteria (18, 19). Although robust data are missing, it appears likely that overdiagnosing might cause an overtreatment of patients after stroke. In our trial, patients in the control group were treated according to the current standards of therapy, where management of infections is based on the clinical judgment of the treating physicians. Current European and US stroke guidelines strictly recommend early antibiotic treatment of poststroke infections but advise against their prophylactic use (8). However, while 36% of patients in PCT group and 37% of patients in control group were diagnosed with a poststroke infection, 63% of patients in the PCT group received antibiotic treatment, compared to 45% in the standard group. With a low threshold for antibiotic treatment after stroke in clinical routine, preventive treatment strategies might not be able to add benefits to patients’ care.

The occurrence of SAP was associated with a twofold increase of mortality within 3 months of follow-up, which is in line with previous studies (20). As there is no evidence of improved outcome by preventing infections so far, there is an ongoing discussion whether a causal relationship between SAP and outcome after stroke exists (5, 21). Caution should be exercised not to label SAP as mere marker of detrimental outcome prematurely, as data available so far do not allow excluding a causal relationship. Although patients in both PASS and STROKE-INF received antibiotics that are commonly used for pneumonia treatment in a prophylactic manner, the incidence of SAP remained unchanged compared to patients treated with standard stroke unit care only. This might be due to late initiation of prophylactic treatment in these trials or inappropriate choice of antibiotics (17); however, SAP might also harbor a greater non-infective inflammatory component than previously thought, which might not be prevented by antibiotic treatment alone (7). As a stroke-induced immunosuppressed state is an important risk factor for SAP, the use of targeted immune modulators might be a promising new treatment approach (22).

Our study has several limitations. Our study has small baseline imbalances for stroke severity in favor of the control and for COPD in favor of the PCT group. More importantly, the low adherence to the PCT guidance might have caused an underestimation of the effects of PCTus-guided antibiotic treatment. In addition, the number of included patients was rather small in comparison with the recent phase III studies on preventive antibiotics. However, we only included severely affected stroke patients who are known to have a higher risk of infections, and the overall infection rate in our trial was 37%, which is higher than in the previously reported trials (5, 6). In addition, variations in antibiotic substances and doses might have influenced our results. The high rate of antibiotic treatment in the control group might have confounded benefits of PCTus-guided antibiotic treatment.

In conclusion, our results do not support the use of PCTus-guided antibiotic treatment of SAP to improve long-term outcome after stroke. However, a *post hoc* analysis of our data affirms that PCTus is a useful biomarker for diagnosis of SAP. The utility of PCTus as a biomarker for the diagnostic setup of SAP needs to be addressed in further trials, e.g., a cutoff level with high positive and negative predictive values needs to be established. Moreover, subsequent trials identifying biomarker patterns for prediction of SAP at stroke onset [among others PCTus, copeptin (23)] might provide the rational basis for biomarker-guided anti-infective immunomodulatory treatment already starting in the hyperacute phase of stroke in patients at risk for SAP.

## Co-Investigators and Study Centers

**Benjamin Hotter** (site investigator), Department of Neurology, Charité – Universitaetsmedizin Berlin (Campus Benjamin Franklin, Campus Charité Mitte, Campus Virchow-Klinikum), Germany; **Center for Stroke Research Berlin Trial Team** (site investigator), Department of Neurology, Charité – Universitaetsmedizin Berlin (Campus Benjamin Franklin, Campus Charité Mitte, Campus Virchow-Klinikum), Germany; **Daniel Peters** (site investigator), Department of Neurology, Unfallkrankenhaus Berlin, Germany; **Daniela Thiem-Martin** (site investigator), Department of Neurology, Vivantes Klinikum Spandau, Berlin, Germany; **Duc Thuan Nguyen** (site investigator), Department of Neurology, Vivantes Auguste-Viktoria-Klinikum, Berlin, Germany; **Georg Walter** (site investigator), Department of Neurology, Vivantes Klinikum Spandau, Berlin, Germany; **Grit Lehmann** (site investigator), Department of Neurology, Unfallkrankenhaus Berlin, Germany; **Jens Offermann** (site investigator), Department of Neurology, Vivantes Klinikum Neukoelln, Berlin, Germany; **Jos Goehler**, Department of Neurology, Charité – Universitaetsmedizin Berlin (Campus Benjamin Franklin, Campus Charité Mitte, Campus Virchow-Klinikum), Germany (site investigator); **Olaf Crome**, Department of Neurology, Vivantes Klinikum Neukoelln, Berlin, Germany (site investigator); **Paul Sparenberg**, Department of Neurology, Unfallkrankenhaus Berlin, Germany (site investigator); **Sebastian Boettcher**, Department of Neurology, Unfallkrankenhaus Berlin, Germany (site investigator); **Stephan Kinze**, Department of Neurology, Unfallkrankenhaus Berlin, Germany (site investigator); Neurovascular Research Laboratory, Institut de Recerca, Hospital Universitari Vall d’Hebrón, Universitat Autònoma de Barcelona, Spain; Department of Neurology, Universitaetsspital Zuerich, Switzerland; Department of Neurology, Klinikum Frankfurt Oder, Germany.

## Ethics Statement

This study was carried out in accordance with the recommendations of the responsible local ethics committees [the ethics committee of the Charité University Berlin (EA1/267/10) for study centers located in Berlin, the ethics committee of the State Medical Association of Brandenburg (AS 30(a)/2011) for the study center located in Brandenburg, the Kantonale Ethikkommission Zuerich (2013-0195) for the study center in Zuerich, and the Hospital Vall d’Hebron Clinical Research Ethics Committee (TFS-ANT-2012-01) for the study center in Barcelona] with written informed consent from all subjects or their legal representatives. All patients or their legal representatives gave written informed consent in accordance with the Declaration of Helsinki.

## Author Contributions

LU: design and conceptualization of the study, acquisition of data, analysis and interpretation of data, drafting of manuscript, final approval of the manuscript to be published, and agreement to be accountable for all aspects of the work. SH, DN, MH, B-MM, FH, IS, G-JJ, JM, AB, MK, and AH: acquisition of data, revision of manuscript for intellectual content, final approval of the manuscript to be published, and agreement to be accountable for all aspects of the work. SE and CD: conceptualization of the study, revision of manuscript for intellectual content, final approval of the manuscript to be published, and agreement to be accountable for all aspects of the work. JW and SH: analysis and interpretation of data, revision of manuscript for intellectual content, final approval of the manuscript to be published, and agreement to be accountable for all aspects of the work. CM and SA: design and conceptualization of the study, revision of manuscript for intellectual content, final approval of the manuscript to be published, and agreement to be accountable for all aspects of the work. AM: design and conceptualization of the study, analysis and interpretation of data, drafting of manuscript, final approval of the manuscript to be published, and agreement to be accountable for all aspects of the work.

## Conflict of Interest Statement

SA has received honoraria for consultancies from BRAHMS GmbH. Thermo Fisher Scientific provided procalcitonin kits at reduced costs. Thermo Fisher Scientific had no involvement in data collection or patient recruitment. All other authors declare no competing interests.
